# Quantification of rifampicin in human plasma and cerebrospinal fluid by a highly sensitive and rapid liquid chromatographic–tandem mass spectrometric method

**DOI:** 10.1016/j.jpba.2012.05.028

**Published:** 2012-11

**Authors:** Abhishek Srivastava, David Waterhouse, Alison Ardrey, Stephen A. Ward

**Affiliations:** Liverpool School of Tropical Medicine, Pembroke Place, Liverpool, United Kingdom

**Keywords:** CSF, cerebrospinal fluid, MTB, mycobacterium tuberculosis, RPT, rifapentine, RIF, rifampicin, TB, tuberculosis, Rifampicin, LC–MS/MS, Human plasma, Cerebrospinal fluid, Validation, Quantitative assay

## Abstract

A highly sensitive and rapid liquid chromatography tandem mass spectrometry (LC–MS/MS) method has been developed to measure the levels of the antitubercular drug rifampicin (RIF) in human plasma and cerebrospinal fluid (CSF). The analyte and internal standard (IS) were isolated from plasma and CSF by a simple organic solvent based precipitation of proteins followed by centrifugation. Detection was carried out by electrospray positive ionization mass spectrometry in the multiple-reaction monitoring (MRM) mode. The assay was linear in the concentration range 25–6400 ng/mL with intra- and inter-day precision of <7% and <8%, respectively. The validated method was applied to the study of RIF pharmacokinetics in human CSF and plasma over 25 h period after a 10 mg/kg oral dose.

## 1 Introduction

Tuberculosis (TB) is one of the most important human bacterial diseases caused by mycobacterium tuberculosis (MTB) commonly affecting the lungs [1]. It is an aggressive disease with one third of the World's population reportedly being infected with MTB [1,2]. It is more common in developing countries, with more than half of the reported cases occurring in Asia [1]. Rifamycins are a group of complex, macrocyclic antibiotics produced by *Amycolatopsis mediterranei* that are one of the most important first line anti-TB drugs [3]. Among them RIF has been used for the treatment of TB, leprosy [4,5], some types of osteomyelitis and endocarditis [6]. The drug is most regularly deployed in the cocktail of drugs used as first-line treatment for TB. RIF inhibits DNA-dependent RNA polymerase in bacterial cells. Although the drug has been in use for many decades there are still questions about the appropriateness of dosage regimes in specific patient groups such as those co-infected with HIV and young children. In order to address these questions there is a need for a suitably selective and sensitive analytical method that is capable of measuring the drug in biological fluids. Several analytical methods are already available for the determination of RIF in biological fluids and pharmaceutical dosage forms, including methods based on HPTLC [7], HPLC [8–16], HPLC following SPE [17] or SBSE [18], UPLC [19], LC–MS/MS [20–23] and MALDI–TOF [24]. In all cases there are some drawbacks associated with existing methods making them unsuitable for satisfactory quantitative analysis in studies that have been designed and implemented as part of an integrated TB clinical pharmacology programme in the Liverpool School of Tropical Medicine. In some cases there is no inclusion of an IS which compromises the robustness of the method [8,15,17], some assays need relatively large volumes of sample and/or lacked sensitivity which limits their usefulness in pediatric studies where sample volumes are severely constrained [12–14] and other assays involve complex or very long extraction procedures thereby compromising throughput [10,11,13,16]. The aim of the work presented here was to develop a rapid and sensitive LC–MS/MS method for the quantification of RIF in human plasma and CSF samples. We have established an exacting, sensitive and reproducible analytical method requiring only a small sample volume and involving quick sample processing as key benefits of this assay for the quantitative analysis of RIF. The developed method was validated according to the FDA guidelines [25].

## 2 Materials and methods

### 2.1 Solvents and chemicals

**Fig. 1:**
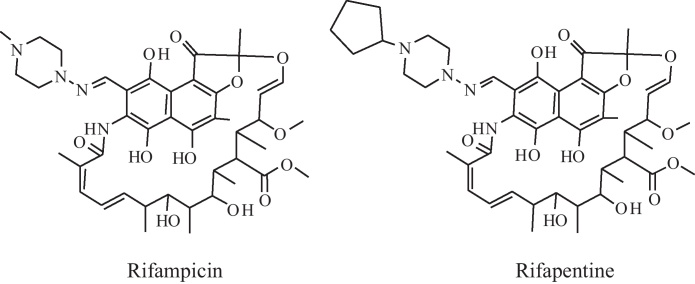
Chemical structures of compounds.

RIF (C_47_H_64_N_4_O_12_; MW 822.94) and rifapentine (RPT; C_15_H_24_O_5_; MW 877.03) were purchased from Sigma–Aldrich (Poole, Dorset, UK). The structures of RIF and RPT are given in Fig. 1. Methanol, water and acetonitrile were all HPLC grade from Fisher Scientific UK (Loughborough, Leicestershire, UK). All other solvents were of HPLC grade and unless otherwise specified all other reagents were purchased from Sigma–Aldrich. Drug free human plasma was obtained from the National Blood Service. Artificial CSF was obtained from Harvard Apparatus (Holliston, MA, USA).

### 2.2 Equipment

The HPLC system consisted of a variable loop Accela autosampler (200 vial capacity set at a temperature of 4 °C) and an Accela LC pump (Thermo Electron Corporation, Hemel Hempstead, UK). A reverse-phase Hypersil–Hypurity C18 column (150 mm × 2.1 mm, i.d., 5 μm; Thermo Electron Corporation, Hemel Hempstead, UK) set at room temperature was used to elute RIF and RPT. The HPLC system was interfaced with a triple-quadrupole TSQ Quantum Access mass spectrometer (Thermo Electron Corporation, Hemel Hempstead, UK) fitted with an electrospray ionization (ESI) source. One E2M30 rotary vacuum pump (Edwards High Vacuum International, West Sussex, UK), a nitrogen generator (Nitro Generator, Products of Technology Ltd., Killearn, UK) and 99% pure argon gas (10 L BIP Gas, Air Products, Crewe, UK) were used.

### 2.3 Standard solutions

RIF and RPT, used as the IS, were weighed from solid to an appropriate amount. Solids were then dissolved in methanol to give 1 mg/mL primary stock solutions. Appropriate dilutions were then made in methanol to produce working stock solutions of 320, 160, 80, 40, 20, 10, 2.5, and 1.25 μg/mL. Another set of working stock solutions was made in methanol (from re-weighed primary stock) at 240, 30 and 3.75 μg/mL for preparation of QC samples accordingly. Aliquots of each working solution were diluted 50 fold with drug-free plasma or CSF to obtain eight calibration standards (CS) and three levels of quality control (QC) samples, namely high (HQC), medium (MQC) and low QC (LQC). CS were made up fresh with each new analytical run whereas QC samples were drawn form a stock of QC samples stored in a −80 °C freezer at the onset of the RIF analytical programme. A working IS stock solution containing 250 ng/mL of RPT was accurately made up (total volume: 400 mL; stored at −20 °C, under dark conditions) by diluting 1 mg/mL stock solution with 50% ACN and 50% methanol. Both RIF and RPT are light sensitive. In order to minimize/eliminate light dependent decomposition all working solutions were stored in the dark and all sample extraction and preparation procedures were performed in a darkened fume cupboard and biological safety cabinet.

### 2.4 Sample preparation

Frozen plasma and CSF samples from study subjects & QC samples were thawed as needed. The same procedure was followed for all samples. 100 μl of each of the CS, QC and test samples was transferred into clean 1.5 mL microfuge tubes. 300 μl of IS (250 ng/mL RPT in 50/50 ACN/methanol) was added and the tubes were vortexed vigorously to allow for maximal protein precipitation. Samples were centrifuged (25 min, 16,200 × *g*) and supernatants (200 μl) transferred to clean glass autosampler vials for LC–MS/MS analysis.

### 2.5 Chromatographic and mass spectrometric conditions

Chromatographic separation was achieved using a rapid step-wise mobile phase gradient operating at a flow rate of 350 μl/min over a total run time of 6 min. The mobile phase consisted of a combination of ACN containing formic acid (0.05%, v/v) and 15 mM ammonium formate buffer (pH 5). A sample aliquot of 50 μl was injected onto the column, and eluted with a gradient of 35–90% ACN from 0 to 4 min, increasing to 95% ACN from 4 to 5 min, and finally reverting to 35% ACN from 5 to 6 min.

Quantitation was achieved by MS–MS detection in positive ionization mode for both RIF and IS. The MS operating conditions were optimized as following: the spray voltage was 4500 V with a tube lens voltage of 124 V and skimmer offset of 0 V. The capillary temperature was set to 275 °C. Nitrogen was used as the sheath gas (40 psi) and auxiliary gas (25 psi). Argon was used as the collision gas at a pressure of 1.5 mTorr (1 Torr = 133.3 Pa). The optimized collision energies for RIF and IS were 10 and 30 eV, respectively. Detection of the ions was performed in the MRM mode using the transitions of *m*/*z* 823.4 → 791.4 for RIF and *m*/*z* 877.4 → 150.8 for RPT (IS), respectively, with a scan time of 0.1 s per transition. TSQ Tune Software (Thermo Electron Corporation, Hemel Hepstead, UK) was used for the automatic optimization of tuning parameters. Data acquisition was performed using Xcalibur 1.3 software (Thermo Electron Corporation, Hemel Hepstead, UK). Peak integration and calibrations were performed using LC Quan™ software (Version 2.5.6, Thermo Electron Corporation, Hemel Hempstead, UK).

### 2.6 Validation of quantitative LC–MS/MS method

The quantitative LC–MS/MS method was validated to determine selectivity, calibration range, accuracy, precision, limit of detection (LOD), limit of quantitation, % recovery, matrix effects, freeze–thaw, autosampler and heat inactivation stability. The initial assay was fully validated for RIF analysis in human plasma according to FDA guidelines. Thereafter a partial validation was carried out for RIF analysis in CSF samples as the only alteration was the change in matrix from plasma to CSF.

#### 2.6.1 Selectivity

The selectivity of the method was evaluated by analysing six independent drug-free plasma samples with reference to potential interferences from isobaric endogenous and environmental constituents.

#### 2.6.2 Calibration curve

Calibration curves were generated to confirm the relationship between the peak area ratios and the concentration of RIF in the standard samples. Fresh CS were extracted and assayed as described above on three different days and in duplicate. Calibration curves for RIF were represented by the plots of the peak-area ratio (RIF/RPT) versus the nominal concentration of the RIF in CS. The line of best fit was generated using 1/concentration^2^ weighted quadratic regression as the mathematical model of best fit. RIF concentrations in QC samples, recovery samples, stability samples and experimental CSF/plasma pharmacokinetic samples were calculated from the resulting area ratio and the regression equation of the calibration curve.

#### 2.6.3 Accuracy and precision

Intra-day accuracy and precision were evaluated by analysis of QCs at four levels (LLOQ, LQC, MQC and HQC; *n* = 6 at each level) on the same day. Inter-day precision and the accuracy were determined by analysing four QC levels on 3 separate days (*n* = 6 at each level) along with three separate standard curves done in duplicates. The accuracy of an analytical method describes how close the mean test results obtained by the method are to the nominal concentration of the analyte. Accuracy was calculated by the following equation, expressed as a percentage:

$$\text{Accuracy}\;(\%)=\frac{\text{mean observed concentration}}{\text{nominal concentration}}\times 100$$

The precision was expressed by coefficient of variation (CV). The CV % indicates the variability around the mean in relation to the size of the mean, and is defined as:

$$\text{CV }(\%)=\frac{\text{standard deviation}}{\text{mean observed concentration}}\times 100$$

#### 2.6.4 Limit of detection and limit of quantification

The lower limit of quantification (LLOQ) was determined based on the criterion that the analyte response at LLOQ is five times baseline noise and it is within the acceptable limit of accuracy and precision. Higher limit of quantification (HLOQ) was assigned to the highest point in the standard curve. Intra-day and inter-day accuracy and precision for HLOQ and LLOQ samples were evaluated in the same manner as the QC samples (Section 2.6.3). The LOD was determined as the lowest concentration which gives a signal-to-noise ratio of three for the analytes.

#### 2.6.5 Carryover test

Carry over tests were performed by injection of blank plasma sample directly after the highest point in calibration curve.

#### 2.6.6 Stability

Autosampler, freeze–thaw and plasma heat-inactivation stability of RIF was determined at low, medium and high QC concentrations. To determine the impact of plasma heat inactivation or freeze–thaw cycles on RIF concentration, samples were heat inactivated for 40 min at 58 °C or underwent 3 freeze (−80 °C) thaw (room temperature) cycles. Following sample treatment/storage conditions, the RIF concentrations were analyzed in triplicates and compared to the control sample that had been stored at −80 °C. Autosampler stability of extracted samples was determined by comparing RIF concentration in freshly prepared samples and samples kept in autosampler at 4 °C for 24 h.

#### 2.6.7 % recovery and matrix effect

Recovery was determined by comparing the area under the curve (AUC) of extracted QC samples (LQC, MQC and HQC) with direct injection of extracted blank plasma spiked with the same nominal concentration of RIF as in the QC samples. This should highlight any loss in signal due to the extraction process. IS recovery was determined for a single concentration of 250 ng/mL.

Matrix effects were evaluated using heat-treated and untreated blank human plasma from six different donors using post-column infusion experiments as described by Hanpithakpong et al. [26]. Post column infusion provides a visual assessment of the effect of the sample matrix over an entire chromatographic run to ensure that no interfering peaks are found in the elution windows of analytes and IS. The syringe pump of the MS was setup containing a mixture of 250 ng/mL RIF and RPT in methanol. This solution was infused directly through a T-connector in to the MS at a constant flow rate of 10 μl/min, whilst the blank plasma extract was injected via the autosampler. The chromatograms produced were compared to an injection of mobile phase. Any ion suppression could be seen as a change in deviation in the response of the infused analyte that was greater than the underlying system noise.

Moreover, estimation of the matrix effects was also obtained by comparing the peak area for samples spiked in elution solution with extracted blank matrix spiked with the same nominal concentration of RIF.

### 2.7 Data analysis

LC–MS data acquisition and processing was performed by LC Quan™ software. Standard curves for quantification of RIF were constructed using a 1/concentration^2^ weighted quadratic regression of the peak area ratio versus RIF concentration. Unknown and QC sample concentrations were back-calculated from the standard curves.

## 3 Results and discussion

### 3.1 Detection and chromatography

**Fig. 2:**
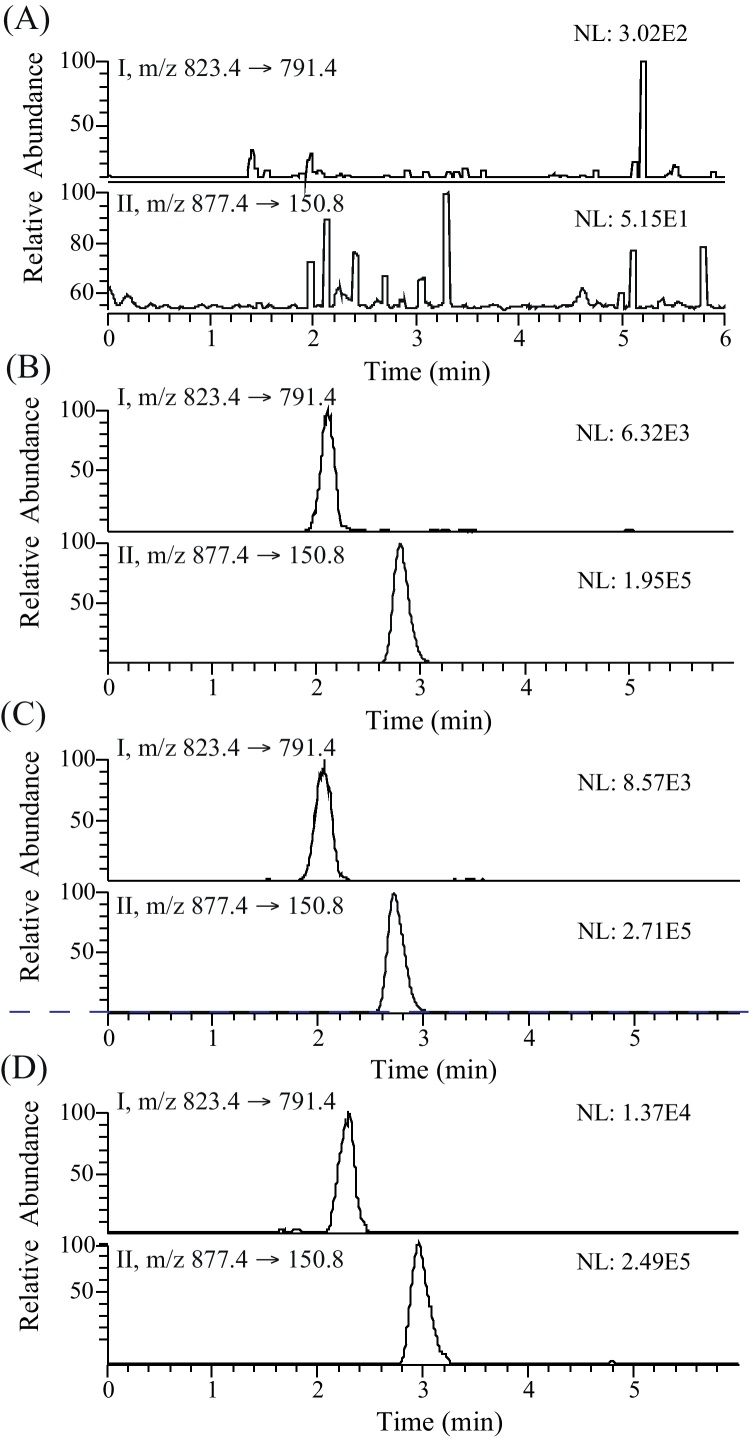
Representative MRM chromatograms of RIF (I) and RPT (II, IS) in: (A) a blank human plasma sample; (B) a blank human plasma sample spiked with RIF at the LLOQ of 25 ng/mL; (C) patient plasma sample 25 h after an oral dose of 10 mg/kg (RIF concentration; 31 ng/mL); (D) CSF sample from a patient receiving a 10 mg/kg daily oral dose of RIF (RIF concentration; 41.83 ng/mL). Sample preparation for 100 μl of (B)–(D) was performed using 300 μl of 50/50 ACN/methanol (including 250 ng/mL IS), whereas IS was not included in case of (A).

Fig. 2 shows the typical chromatograms of a blank human plasma sample (A), a spiked plasma sample with RIF (5.0 ng/mL, LLOQ) and IS (250.0 ng/mL) (B), a plasma sample from a patient 25 h after a 10 mg/kg oral dose of RIF (C) and a CSF sample from a patient receiving a 10 mg/kg daily oral dose of RIF (D). The retention times for RIF and IS were 2.1 and 2.8 min, respectively.

### 3.2 Method validation

#### 3.2.1 Selectivity

The method was found to have high selectivity for the analytes, since no interfering peaks from endogenous compounds were observed at the retention time for RIF or RPT in any of the six independent blank plasma extracts evaluated (Fig. 2A).

#### 3.2.2 Calibration curves

Calibration curves for RIF in human plasma and CSF were fitted by weighted 1/concentration^2^ quadratic regression, with the *r*^2^ values of >0.99 for all curves generated during the validation and pharmacokinetic plasma analysis. The calibration curve accuracy for plasma is presented in Table 1 demonstrating that measured concentration is within ±15% of the actual concentration point (20% for the lowest point on the standard curve, the LLOQ). Results were calculated using peak area ratios. (Calibration curve data for CSF not shown)

#### 3.2.3 Accuracy and precision

A detailed summary of the intra-day and inter-day precision and accuracy data generated for the assay validation is presented in Table 2. Inter-assay variability was expressed as the accuracy and precision of the mean QC concentrations (LLOQ, LQC, MQC, and HQC) of three separate assays. Intra-assay variability was determined as the accuracy and precision of the six individual QC concentrations within one assay. The inter- and intra-assay accuracy and precision was <10% for all QC concentrations, which was within the general assay acceptability criteria for QC samples according to FDA guidelines [25].

#### 3.2.4 Limit of detection and limit of quantification

LOD was defined as the lowest concentration that produced a peak distinguishable from background noise (minimum ratio of 3:1). The approximate LOD was 2.5 ng/mL. The LLOQ has been accepted as the lowest points on the standard curve with a relative standard deviation of less than 20% and signal to noise ratio of 5:1. Results at lowest concentration studies (25 ng/mL) met the criteria for the LLOQ (Table 2). The method was found to be sufficiently sensitive for the determination of RIF in human plasma samples and CSF. The HLOQ has been accepted as the highest points on the standard curve with a relative standard deviation of less than 15% [25].

#### 3.2.5 Carryover test

A critical issue with the analysis of many drugs is their tendency to get absorbed by reversed phase octadecyl-based chromatographic packing materials, resulting in the memory effect. However in this analysis no quantifiable carryover effect was obtained when a series of blank (plasma) solutions were injected immediately following the highest calibration standard.

#### 3.2.6 Stability studies

The results of autosampler, freeze–thaw and heat-inactivation stability are presented in Table 3. Determination of RIF stability following three freeze–thaw cycles showed that for all QC samples there was a minor change in the RIF concentration. In contrast heat-inactivation showed major impact on the RIF concentrations measured for LQC (20.9%) and MQC (22.4%). However, HQC only showed a difference of 5.4% from initial concentration.

#### 3.2.7 % recovery and matrix effect

Percentage recovery of RIF was measured by dividing the AUC values of extracted QC samples with direct injection of solution containing the same nominal concentration of compounds as the QC samples in extracted blank plasma. RIF recovery from plasma spiked with 75, 600, and 4800 ng/mL of RIF was 92.5 ± 2.17, 93.2 ± 3.04 and 94.0 ± 2.70, respectively (*n* = 6). IS recovery at 250 ng/mL of RPT was 96.93 ± 2.39.

**Fig. 3:**
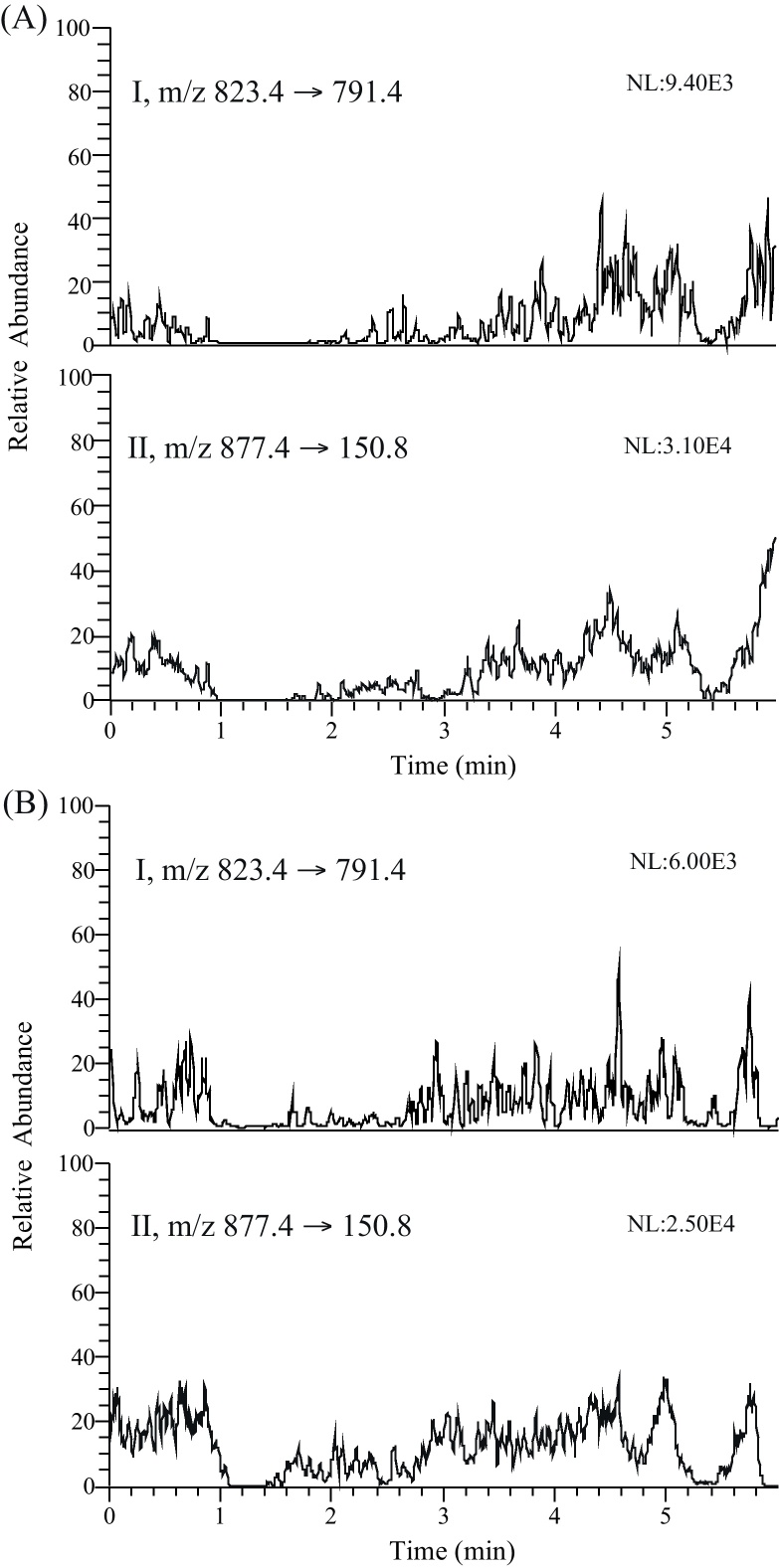
MRM chromatograms obtained after injection of extracted (A) heat-treated and (B) untreated blank human plasma during post column infusion of RIF (I) and RPT (II). A mixture containing 250 ng/mL RIF and RPT in methanol was infused directly by syringe pump through a T-connector in to the MS at a constant flow rate of 10 μl/min, whilst the blank plasma extract was injected via the autosampler.

Post-column infusion experiments confirmed the absence of regions with severe matrix effects (i.e. no sharp drops or increases in the response) for heat-treated and untreated blank human plasma extracted with the developed method (Fig. 3). Moreover, no suppression/enhancement for RIF could be detected when compound in neat injection solvent was compared with compound in extracted blank biological matrix.

#### 3.2.8 Analysis of clinical samples

This method has been used for the measurement of RIF concentration in patient plasma and CSF samples (Table 5). Plasma-concentration data was analyzed by non-compartmental analysis using the Kinetica software package (Thermo Electron Corporation, UK) to obtain pharmacokinetic parameters (Table 4) including area under the curve (AUC_0–*t*_) with extrapolation to infinity (AUC_0–∞_) and apparent elimination half-life (*t*_1/2_) values. Maximum plasma concentration (*C*_max_), the time-to-maximum concentration (*T*_max_) values were obtained by visual inspection of the plasma concentration–time profile.

## 4 Conclusion

An LC/ESI–MS/MS method was developed and validated for the determination of RIF in human plasma. The sample pre-treatment was a single-step liquid–liquid extraction procedure without any requirement for a drying step for sample concentration. Usually drying and reconstitution step is used to obtain lower sensitivity but the present method directly gives sensitivity as low as 25 ng/mL, further we can still go lower by adding drying and reconstitution step to the current method. This assay requires only a small volume of plasma (100 μl). This may be of particular advantage for pediatric studies where children can only provide small volumes of blood, and when studying RIF concentrations in the CSF where sample volumes are limited. MTB is generally not present in large amounts in human plasma; however, the potential of HIV and/or hepatitis co-infection means that plasma samples need to be heat inactivated (58 °C, 40 min), however, data shown here indicate that the process of heat-inactivation significantly affects the size of the RIF signal. Therefore, we recommend the omission of heat inactivation where possible.

In conclusion, method validation following FDA guideline indicated that the developed method had high sensitivity with an LLOQ of 25 ng/mL, high recovery (>90%), reliability, specificity and excellent efficiency with a total running time of 6.0 min per sample, which is important for large batches of samples. The sensitive, simple and rapid LC/MS/MS assay is suitable for pharmacokinetic, bioavailability or bioequivalence studies of RIF in human subjects. This method has been successfully applied to analyze RIF concentrations in human plasma and CSF.

## Figures and Tables

**Table 1:** Analysis of plasma CS.

	Nominal conc. (ng/mL)	Mean conc.^a^ (ng/mL)	CV (%)	Accuracy (%)
CS-1	25.00	24.48 ± 2.0	8.15	97.91
CS-2	50.00	49.42 ± 3.8	7.68	98.83
CS-3	200.00	198.65 ± 14.27	7.19	99.33
CS-4	400.00	426.72 ± 9.44	2.21	106.68
CS-5	800.00	793.48 ± 78.86	9.94	99.19
CS-6	1600.00	1550.05 ± 76.84	4.96	96.88
CS-7	3200.00	3250.46 ± 227.62	7.00	101.58
CS-8	6400.00	6377.16 ± 205.98	3.23	99.64

a Data represent the mean ± S.D of six observations.

**Table 2:** Intra- and inter-day precision and accuracy data for assays of RIF in human plasma.

	Inter-day (*n* = 18)			Intra-day (*n* = 6)		
	Mean conc.^a^ (ng/mL)	CV %	Accuracy %	Mean conc. (ng/mL)	CV %	Accuracy %
LLOQ (25 ng/mL)	23.23 ± 1.34	5.78	92.93	24.64 ± 1.51	6.16	98.58
LQC (75 ng/mL)	79.07 ± 6.13	7.75	105.43	82.59 ± 1.52	1.85	110.12
MQC (600 ng/mL)	641.33 ± 37.74	5.88	106.89	649.10 ± 16.81	2.59	108.18
HQC (4800 ng/mL)	5156.19 ± 268.85	5.21	107.42	4846.14 ± 109.91	2.27	100.96
HLOQ (6400 ng/mL)	6610.76 ± 223.44	3.38	103.29	6590 ± 372.57	5.65	102.98

a The inter- and intra-assay data represent the mean ± S.D. of 18 and 6 observations, respectively.

**Table 3:** Stability of RIF in extracted sample and human plasma.^a^

Treatment	LQC	MQC	HQC
Extracted sample/autosampler	4.0	−2.5	−0.8
Plasma/heat- inactivation	20.9	22.4	5.4
Plasma/3 × freeze–thaw	−5.3	2.0	4.6

a Data represented as % difference from initial sample.

**Table 4:** The main pharmocokinetic parameters of RIF in human plasma after an oral dose of 10 mg/kg.

Parameter	Values^a^
*C*_max_ (ng/mL)	3769
*T*_max_ (h)	4.0
*t*_1/2_ (h)	2.8
AUC_0–*t*_ (ng h/mL)	27822.9
AUC_0–∞_ (ng h/mL)	27948.8

a Data presented here is from one patient only.

**Table 5:** Quantification of RIF in human CSF after an oral dose of 10 mg/kg/day.

Sample ID	RIF concentration (ng/mL)
Patient 1 day 0	BLQ^a^
Patient 1 day 3	28.78
Patient 1 day 6	39.49
Patient 1 day 27	30.62
Patient 1 day 62	41.83
Patient 1 day 83	25.59
Patient 2 day 0	BLQ
Patient 2 day 55	96.89
Patient 2 day 83	268.84

a BLQ, below limit of quantification.
